# Study of lipase production by a filamentous fungus isolated from soil contaminated with lipid residues

**DOI:** 10.1186/1753-6561-8-S4-P171

**Published:** 2014-10-01

**Authors:** Milena Zardetto Meloni, Mariana Ribeiro Benfatti, Cristiani Baldo, Fabiana Guillen Moreira-Gasparin, Maria Antonia Pedrine Collabone Celligoi

**Affiliations:** 1 Universidade Estadual de Londrina, Londrina, PR, Brazil

## 

Lipases are the special kind of esterases belong to subclass 1 of hydrolytic enzyme class 3 and have been assigned sub-sub class 3.1.1 due to their specificity for carboxylic acid ester bonds. The biological function of lipase is to catalyze the hydrolysis of triacylglycerols to give free fatty acid, diacylglycerols, mono-acylglycerols and glycerol. They constitute the most important group of biocatalysts for biotechnological applications. Furthermore, novel biotechnological applications have been successfully established using lipases for synthesis of biopolymers and biodiesel, production of enantiopure pharmaceuticals, agro-chemicals and flavour compounds. The aim of the present study was to evaluate the production of lipases by filamentous fungi isolated from soil in different growing conditions. The lipase production was evaluated by both solid-state with soybean meal and submerged fermentation with different carbon and nitrogen sources. In addition, different concentrations of olive oil and ammonium nitrate and temperature were evaluated through a full factorial design (3^3^) with three replications at the center point. All submerged fermentations were shaken (120rpm). Lipase activity in the culture filtrate was determined spectrophotometrically at 37°C using p-nitrophenol palmitate (pNPP) as substrate. One enzymatic unit (1U) was defined as that amount of enzyme that liberated 1µmol of *p*NPP per minute under the test conditions. Except for the fermentation at different temperatures, all experiments were carried out at 28°C. Olive oil and ammonium nitrate were, respectively, the best carbon and nitrogen sources. Low lipase activity was found in the solid-state cultures, which is interesting, since soybean meal was used without inducing lipid supplementation. In the factorial design, analysis of variance (ANOVA) showed significant effects when temperature and oil concentration varied. The maximum level of lipase production (206.68U/ml) was reached with 0.5%(W/V) of olive oil, 1.5%(W/V) of ammonium nitrate and at the temperature of 24°C. Lipase production is generally stimulated by lipids and is usually coordinated with the availability of triglycerides. Furthermore, nitrogen sources in the medium have to be carefully considered for growth and optimization of production. Response surface methodology has been widely used to bring insights of the interaction effects of several process parameters, generally resulting in higher production yields. In this work, with the evaluated levels of each tested parameter, we did not achieve an optimized condition. Further experiments are needed in order to get an optimized process of lipase production. Nevertheless, the soil-isolated filamentous fungi demonstrated an interesting ability to produce lipase in both solid-state and submerged fermentation.
